# The comparison of roof visibility of the mandibular canal between cone-beam computed tomography scans and panoramic radiograph images as dependent on the cortical bone thickness of the mandible

**DOI:** 10.1186/s40729-021-00324-z

**Published:** 2021-05-18

**Authors:** Ali-Reza Ketabi, Angeliki Zelka, Hans-Christoph Lauer, Stefan Hassfeld

**Affiliations:** 1 Private Practice, Epplestr. 29 A, 70597 Stuttgart, Germany; 2 Karlsruhe, Germany; 3 Department of Prosthodontics, Center for Dentistry and Oral Medicine (Carolinum), Johann Wolfgang Goethe-University, Theodor-Stern-Kai 7, 60596 Frankfurt, Germany; 4 Department of Oral and Maxillofacial Surgery, Dortmund Hospital gGmbH and Faculty of Health, Witten/Herdecke University, Münsterstr. 240, 44145 Dortmund, Germany

**Keywords:** Mandible canal, CBCT, Diagnostic imaging, Panoramic radiography, Inferior alveolar nerve

## Abstract

**Background:**

Accurate detection of the mandibular canal is a difficult process despite cutting-edge radiographic methods. The present study analyses whether mandibular canal roof visibility is comparable to panoramic radiography (PR) and cone-beam computed tomography (CBCT) and, further, examines whether the visibility in PR and CBCT is dependent on cortical bone thickness in the mandible.

**Methods:**

This study was conducted on a group of 343 selected patients. It incorporated anonymised data on 343 patients in which a CBCT and PR were available. The first stage examines whether the mandibular canal roof visibility is comparable to PR and CBCT. In the second stage, measurements of cortical bone thickness showed buccal and lingual in the P2, M1, M2 and M3 teeth areas, both to the left and right of the mandible in CBCT images.

Statistical analysis was supported by statistical software (IBM SPSS 25; Armonk, NY, USA).

**Results:**

The mean age of the patients was 58.8 years with an almost equal gender distribution. When performing a McNemar test on the P2, M1, M2 and M3 on both the left and right jaws, the difference between the two image modalities, with regard to the visibility of the canal roof, was found to be significant (McNemar test, *p* < 0.001).

Statistically (*U* test, *p*≥0.05), it follows that the thickness of the cortical bone of the mandible exerts no influence on the visibility of the roof of canalis mandibulae in PR and CBCT images.

**Conclusion:**

We conclude that the visibility of the mandibular canal in PR and CBCT rays is not identical, and that the thickness of the cortical bone in the mandible does not represent a factor affecting the visibility of the roof of the mandibular canal.

## Background

One essential pre-requisite for a successful surgical intervention in the lower jaw is precise knowledge of the shape of the mandibular canal. This is of significance for implant-related questions, but no less for augmentations, the surgical removal of wisdom teeth or apicoectomy.

In dental diagnostics, panoramic radiographs (PR) have remained the gold standard for several decades [1]. The introduction to dentistry of cone-beam computed tomography (CBCT) in 1998 facilitated three-dimensional imaging of the head area [2]. In diagnostic dentistry, this has allowed for precise situational assessment and for the non-overlapping visualisation of anatomical structures. Consequently, CBCT is of major clinical relevance in dental diagnostics.

Scientific studies evaluate differently the question as to whether the three-dimensional CBCT recording technique is superior to that of two-dimensional panoramic recording. Literature on the subject comprises numerous comparative studies on the diagnostic value of CBCT and PR in several fields of dentistry [3, 4].

The evaluative scope of the canalis mandibulae—above all, with respect to the visibility of the canal roof—in PR as opposed to CBCT, has yet to be researched comprehensively.

The objective of the present study is the comparative evaluation of the diagnostic value of two-dimensional and three-dimensional radiographs by means of the recognisability of the roof of the canalis mandibulae. Here, the guiding hypothesis is that the roof of the canalis mandibulae is equally identifiable in both two- and three-dimensional images. Should the hypothesis be disproven, and the CBCT of a two-dimensional image and the detectability of the roof of the mandibular canal prove superior, this will, in turn, give rise to the further question as to whether the thickness of the buccal and oral cortical bone of the mandible represents an influencing factor on visibility.

## Methods

The researcher was briefed by an expert in the field of dental radiology prior to commencement of the study. The reliability study was conducted following radiographic instructions and under standardised conditions (<1000 lux) on an accredited diagnostic monitor (EIZO FlexScan S2000 1024×1280 pixels). The measurements were carried out for a maximum of 6 h per day with a 30-min break at 2-h intervals. The results were analysed for reliability.

To verify the reliability of radiographic measurements and evaluations, multiple ratings were carried out on a total of twenty, randomly selected patients. To assess inter-rater reliability, these were then evaluated by the researcher and the expert. For testing the intra-rater reliability, the same images were reviewed a second time by the same expert following a 2-week interval. The findings were found to fully concur. The intra-rater reliability as well as the inter-rater reliability proved to be very high, with a Cohen’s kappa of 1.0 and a 95% confidence interval for kappa [0.92; 1.00]. To verify the intra-rater reliability, these images were evaluated a second time by the same assessor after a 2-week interval. The findings indicated complete concurrence. Both intra-rater and inter-rater reliability proved to be very high with a Cohen’s kappa of 1.0 and a 95% confidence interval for kappa [0.92; 1.00].

For the present study, 549 patients were initially selected from the database of a dental practice in Stuttgart, Germany, between February 2010 and January 2017, whereby both a panoramic image and a CBCT image were already available prior to the commencement of the study.

The following inclusion criteria were applied for the inclusion of patients in the study:

- Patients with both PR and CBCT images in the mandible
- Images with correct patient positioning
- No artefacts in the measurement area of the mandibular canal roof

Thus, the number of patients who fulfilled the inclusion criteria was reduced to 343. The admissions were conducted according to different justifying indications and independently of the study.

As part of the study, patient data was anonymised and X-ray images consecutively numbered. Thus, this ruled out the possibility of allocation of data to patient.

The mapping of the existing jaw sections in the CBCT images and panoramic slice images were audited prior to the definitive evaluation. In the case of many patients, for example, the field of view (FOV) in the CBCT image was smaller than the PR, such that in these patients not all regions could be accounted for. Areas that were missing, either in CBCT or PR images, were duly noted and excluded from the study.

In the present study, panoramic radiographs were taken with the Orthophos D 3297 X-ray unit (Sirona dental Systems GmbH, Bensheim, Germany) and recorded on an imaging plate (Vistascan View, Dürr Dental, Bietigheim-Bissingen, Germany). The exposure parameters were 60 KV, 10 mA, and 16.4 s. This was read out by means of an imaging plate scanner (Vistscan Combi Plus, Dürr Dental). The evaluation was carried out using professional imaging software DBSWin (version 5.1.1; Dürr Dental SE, Bietigheim-Bissingen, Germany).

The CBCT images were obtained by a Gendex CBX-500™ (KaVo Dental GmbH, Biberach, Germany). The acquisition parameters were 90 kV, 8.9 s with a 0.3-mm resolution. The images were evaluated with the Viewer-Software i-cat Vision (Imaging Sciences International, Hatfield, PA, USA).

The measurements were obtained under standardised conditions in a darkened room of room class 5 (<1000 lux) on a diagnostic monitor (Eizo Flex Scan S2000).

For diagnostic purposes, the mandible was divided into the following sections: P2 second premolar, M1 first molar, M2 second molar, and M3 third molar each on the left and right. Thus, up to eight different regions were assessed per jaw and per exposure.

**Fig. 1:**
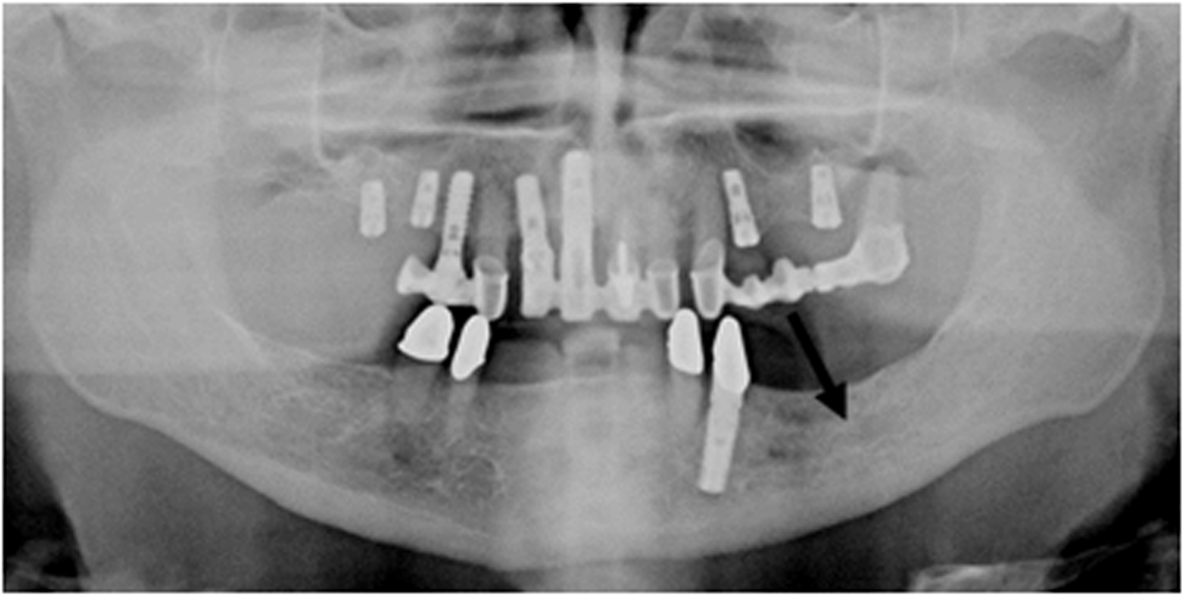
PR: region 36 canal roof not visible

**Fig. 2:**
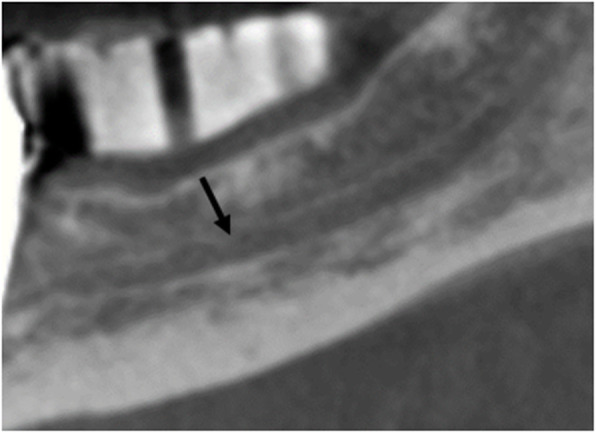
CBCT: region 36 canal roof visible

**Fig. 3:**
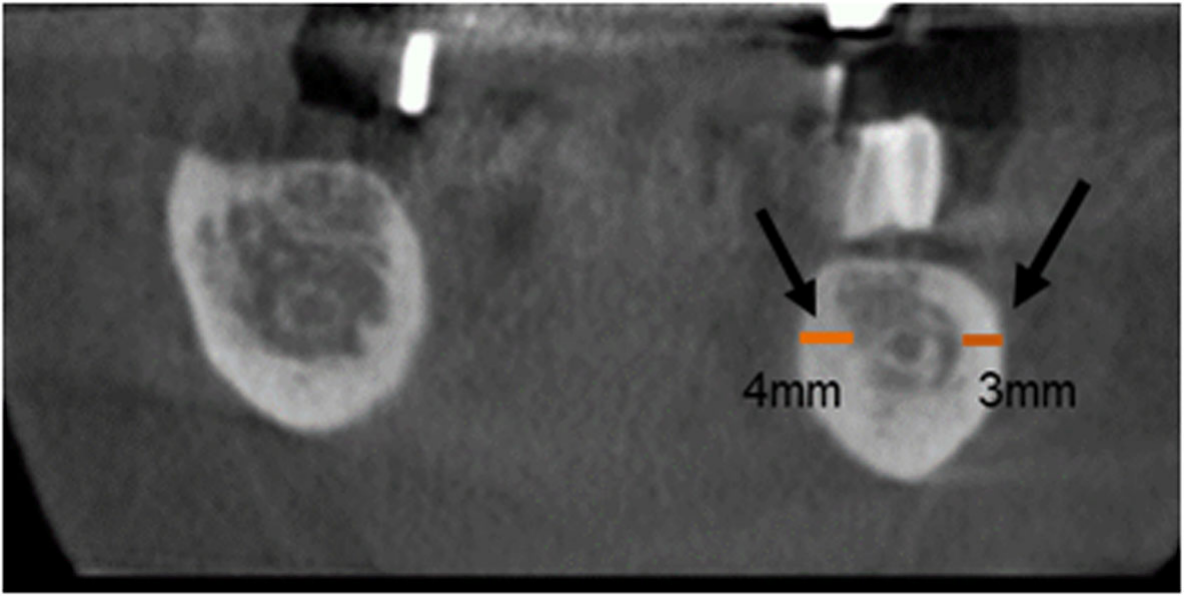
Section of CBCT image of region 36 with buccal and lingual measurement of cortical bone thickness

Both the PR images (Fig. 1) and the CBCT images (Fig. 2) were analysed with reference to the visibility of the roof of the canalis mandibulae. Furthermore, in the CBCT images, the thickness of the buccal and oral cortical bone of the respective regions P2-M3 was measured at the level of the roof of the mandibular canal (Fig. 3).

Excel 2016 (Microsoft Inc., Redmond, Washington, USA) was used to process patient data and X-ray indications. The first step consisted in evaluating all panoramic slice images. This was followed by the corresponding reporting of the CBCT image. The diagnostic parameters were determined by evaluating 57 publications researched by means of a Pubmed search and which were deemed relevant for the purpose of these studies.

The findings were entered into a customised mask developed by the Institute for Statistics (MediStat GmbH, Kornshagen, Germany) and analysed by Ulrike von Hehn (medistat GmbH, Kronshagen, Germany) using the SPSS Statistic 25 software (IBM Corporation, Armonk, New York, USA). The CBCT and PR measurements were tested by the Wilcoxon test for pair differences for variations. Two independent samples were compared by means of the Mann and Whitney *U* test. To examine correlations between quantitative, not normally distributed parameters, a rank correlation analysis was carried out as per Spearman.

The McNemar test and the chi-square test were used for a statistical testing of the working hypothesis (differences between imaging techniques regarding the visibility of the roof of the canalis mandibulae). A *p* value of ≤ 0.05 indicates the presence of a significant difference.

## Results

The cohort of 343 patients numbered a total of 174 women (50.7%) with an average age of 59.1 years, whilst the 169 men (49.3%) were of an average age of 58.8 years. The justifying indications were distributed as follows: 223 implant planning (69.0%), 58 prior to surgical wisdom tooth removal (18.0%), 21 endodontic- (6.5%) and periodontal (6.5%)-related issues respectively.

Within the scope of the evaluation, some cases could not be evaluated due to input errors during the examination. These 20 cases (5.8% of the cases) were not included in the evaluation; the statistical analysis is consequently based on 323 patients.

### Visibility of the roof of the canalis mandibulae

Table 1 details the visibility of the canal roof in the different tooth regions (P2-M3) in the PR and CBCT images. Initially, it had been shown that visibility in all regions examined was significantly higher in the CBCT than in the PR (Table 1).

**Table 1:** Visibility of the roof of the CM in the PR versus CBCT

	Visibility in PR	Visibility in CBCT	Visibility only in CBCT	No visibility in CBCT and PR	Total (significance)
**P2**					
Left	39.2% (125)	100% (319)	60.8% (194)		319 (*p*<0.001)
Right	48.6% (156)	100% (321)	51.4% (165)		321 (*p*<0.001)
**M1**					
Left	12.9% (41)	100% (319)	87.1% (278)		319 (*p*<0.001)
Right	13.4% (43)	100% (321)	86.6% (279)		321 (*p*<0.001)
**M2**					
Left	14.7% (47)	100% (319)	85.3% (273)		319 (*p*<0.001)
Right	12.5% (40)	100% (320)	87.5% (281)		320 (*p*<0.001)
**M3**					
Left	28.2% (89)	96.8% (306)	68.7% (217)	3.2% (10)	316 (*p*<0.001)
Right	37.5% (118)	96.2% (303)	58.7% (185)	3.8% (12)	315 (*p*<0.001)

Visibility of the CM in the PR and CBCT in the individual jaw sections P2, M1, M2 and M3 respectively, left and right in percent (absolute)

Since the selected FOV of the CBCT image was to be as small as possible, P2-M2 was partially visible and M3 no longer visible, or conversely, M1-M3, but not P2. This explains why the sums do not result in the expected 323 recordings taken.

The roof of the canalis mandibulae is best visible in the PR in the region of the 2nd premolar (39.2% left and 48.6% right). However, in many cases, it cannot be identified in the region of the first molar (12.9% left and 13.4% right) and second molar (14.7% left and 12.5% right). In the area of the 3rd molar (28.2% left and 37.5% right), the canal roof is again significantly more identifiable.

The roof of the mandibular canal was visible in all CBCT images in regions P2-M2, in region M3 exceeded 96.8% on the left and 96.2% on the right. In 10 CBCT images on the left (3.2%) and 12 images on the right (3.8%), the canal roof could not be detected.

There was no case in which the canalis mandibulae was visible in the PR but not in the CBCT.

As already evident from the *p* values, the McNemar test showed a significant distinction between the two image modalities (McNemar test, *p* < 0.001) in terms of the visibility of the canalis mandibulae in all regions (Table 1). Consequently, the zero hypothesis, stating that the canalis mandibulae is equally recognisable in both two- and three-dimensional images, may be rejected.

### Influence of the thickness of the cortical bone on the visibility of the roof of canalis mandibulae

In this study, the comparative measurements of the thickness of the cortical bone were based on the visibility of the canalis mandibulae. To this end, the findings were divided into two groups.

1. Group: The visibility of the roof of the canal in PR and CBCT images was identical: CBCT=PR
2. Group: The roof of canalis mandibulae was visible in CBCT images, but were not visible in PR images: CBCT≠PR

Table 2 illustrates the average values of the compact thickness of the cortical bone in the CBCT=PR and CBCT≠PR group (Table 2).

**Table 2:** Visibility and discrepancy of the CM in PR versus CBCT

P2		*N*	CBCT=PR	*N*	CBCT≠PR
Left	Lingual	125	2.05	193	2.04
	Buccal	125	2.25	193	2.18
Right	Lingual	155	2.10	165	2.15
	Buccal	155	2.31	165	2.31
M1		*N*	CBCT=PR	*N*	CBCT≠PR
Left	Lingual	41	1.86	277	1.96
	Buccal	41	2.50	277	2.42
Right	Lingual	42	1.89	279	2.01
	Buccal	42	3.10	279	2.48
M2		*N*	CBCT=PR	*N*	CBCT≠PR
Left	Lingual	46	1.54	273	1.74
	Buccal	46	2.64	273	2.66
Right	Lingual	39	1.80	281	1.80
	Buccal	39	2.72	281	2.73
M3		*N*	CBCT=PR	*N*	CBCT≠PR
Left	Lingual	89	1.69	217	1.69
	Buccal	89	2.52	217	2.59
Right	Lingual	118	1.69	185	1.68
	Buccal	118	2.63	185	2.53

Average values of the thickness of the cortical bone (in mm) in the individual jaw sections P2, M1, M2 and M3 respectively left and right as well as buccally and lingually, with consistent visibility of the CM (CBCT=PR) and with discrepancy (CBCT ≠PR)

For technical reasons (e.g. artefact formation), the thickness of the cortical bone could not be measured in all images. Therefore, the sum of the measured values in the lines is not obtained in the expected value of 323 recordings.

In region M1, where the canalis mandibulae is detected considerably less frequently in the PR, the thickness of the cortical bone in such cases in which visibility in the CBCT and PR are equal, is 1.86 mm left lingually, and 2.50 mm buccally, and 1.89 mm right lingually and 3.1 mm buccally. In the group with divergent visibility, the compact thickness is left lingually 1.96 mm, and buccally 2.42 mm, and right lingually 2.01 and buccally 2.48.

In the second premolar P2 region, the canalis mandibulae is most visible in the PR. Here, in the group CBCT=PR, the thickness of the cortical bone region P2 was 2.05 mm lingually and 2.25 mm buccally on the left, and 2.10 mm lingually and 2.31 mm buccally on the right. In the CBCT≠PR group, the value was 2.04 mm lingually and 2.18 mm buccally on the left and 2.15 mm lingually and 2.31 mm buccally on the right.

When evaluating the results, it is evident that in both cases—regardless of whether the canal roof was visible only in the CBCT or in the PR and CBCT—the measured values of the compact thickness remain at a comparable level. The table indicates no clear difference in the measurements.

Statistically (*U* test, *p*≥0.05), it follows that the thickness of the cortical bone of the mandible exerts no influence on the visibility of the canalis mandibulae in PR and CBCT images.

## Discussion

### Review of methods

At the outset of the study, 549 patient records, which were compiled in the period 2010-2017 in a private dental practice in Stuttgart, were selected and anonymised as a first step. The decisive selection criteria were the availability of a PR and CBCT image of the patient without artefacts in the measurement area, and correct patient positioning. Following the data analysis, it was found that only 343 patients fulfilled the selection criteria with the result that only the latter were included in the present study. In addition, a further 20 cases, which were not evaluable due to input errors, could not be accounted for in the statistical evaluation. Despite this, in comparison to similar studies, the remaining 323 (174 women of an average age of 59.1 years and 169 men with an average age of 58.8 years) patients represent a high number of admissions used for statistical analysis [5–7].

The measurements were conducted in a darkened room under standardised conditions and on an approved diagnostic monitor. Due diligence was taken to minimise the risk of measuring errors by confining the work to a maximum of 6 h and allowing for regular breaks at intervals of 2 h. In studies dealing with similar questions, this is not the case [8, 9].

The reliability of the measurements was verified by an expert in a preliminary study. Following the completion of the measurements, which were repeated at 2-week intervals, these data were then submitted to the Medistat company for static evaluation. The intra-rater reliability of the principal investigator as well as the inter-rater reliability between the principal researcher and the expert was very high, with a Cohen’s kappa of 1.0 and a 95% confidence interval for kappa [0.92; 1.00].

For this study, there was only one researcher who initially evaluated the panoramic images before evaluating the CBCT images after a minimum of 2 weeks. A higher statistical significance may have been obtained had several examiners been involved in the study. However, the high intra- and inter-rater reliability (Cohen’s kappa 1.0) of the preliminary study indicates the reliability of results.

One further influence could be the differentiation of two classes as applied in the method: the roof of the canalis mandibulae ‘visible’ or ‘not visible’. This led to the classification ‘not visible’ in a large number of PR images. The use of a method with multiple differentiation possibilities may have yielded a different result in contrast to the one obtained here. In their study, Peker et al. [10] compared the quality of conventional and digital radiographs on a three-point scale in terms of anatomical structures and pathological findings. Statistically, their findings indicate equivalence in the quality of both digital and conventional radiographs. Consequently, no further differentiation would appear to merit further consideration as this represents no clinical relevance. Furthermore, from a clinical standpoint and with respect to diagnostic utility, only the unambiguous statement ‘visible’ or ‘not visible’ is of relevance.

The diagnostic images were analysed on a tooth-by-tooth basis. Thus, the statistical evaluation was also tooth-related, and the respective findings were specified for the regions on the right and on the left. As anticipated, only very small lateral differences were detected following data evaluation. This demonstrates that the process of data acquisition was carried out meticulously.

### CBCT versus PR visibility

On comparing the two methods, Angelopoulos et al. [5] found that CBCT images yielded superior results in terms of detectability (irrespective of region) of the canalis mandibulae.

In their study, Jung et al. [6] examined the path and visibility of the canalis mandibulae in the PR and in the CBCT in 262 patients. Their findings showed that 22.7% of the canalis mandibulae in the first molar region (M1) was not visible in the PR image. In the area of the second molar M2, the canal was not visible in 11.8% of cases, whilst in the area of the wisdom tooth M3, it was not visible in only 1.3% of cases. As anticipated, visualisation of the canalis mandibulae was superior in the CBCT images, whereas in the M1 region visualisation was absent in only 8.2% of the cases. In the region of the second molar it was unidentifiable in only 5.7% of cases, and indeed, in the region of the third molar, a mere 0.2% of cases.

In the study by Valedec et al. [7], the bone thickness vestibular of the canalis mandibulae was measured in 314 CBCTs. Region P2 exhibited an average bone thickness of 4 mm. At M3 level, it increases further distally to 6 mm and decreases again to 3 mm. For detailed planning of surgical intervention, Valedec recommends the preparation of a CBCT to determine the horizontal bone thickness vestibular of the CM. However, possible influence of bone thickness on CM visibility was not investigated. Not unlike the studies carried out by Angelopoulos et al. and Jung et al., the results of this study demonstrate that the roof of the canalis mandibulae is evidently more frequently visible in CBCT images than the case in PR.

In the present study, however, in contrast with the abovementioned studies [5–7], a more pronounced discrepancy was observed in the visibility of the canal roof between the CBCT and the PR. One plausible cause of this could be the use of imaging plates. This may have had an adverse effect on the visibility of the canal roof in PR and consequently on the results. However, in their study, Baksi et al. [11] report that for the recognition of anatomical structures, film and unfiltered panoramic images based on imaging plates perform equally well with respect to overall quality. The use of imaging plates in this study is therefore expected to exert a relatively minor influence on the results.

As indicated in the above discussion, the roof of the CM seems to be the most difficult to identify in the area of the first molar [6, 12, 13]. As with the studies cited, in our study, visibility of the CM roof was lowest in the area of the first molar, whereby visibility gradually increased towards the third molar (Table 1).

Few studies have treated the various factors that influence the visibility of the canal roof. A review of literature on the subject reveals interesting and controversial results.

Radiological images show the CM as a hypodense structure surrounded by an upper- and lower-corticated sheath which may differ according to case [14]. This is perhaps why in many cases the boundaries of the CM unrecognisable [5, 13, 15–17]. In their study, de Oliveira-Santos et al. [18] observed on CBCT cross-sectional images the corticalisation of the mandibular canal of the first molar region. They determined that the CM is also clearly visible in the non-corticalised areas.

A similar conclusion is reached both by Wadu et al. [17] and Kubilius et al. [19] in their studies, in which they examined the visibility of the boundaries of the CM morphometrically and densiometrically. The visibility of superior and inferior CM boundaries was not related to the morphometric or densitometric assessment parameters.

Some authors have investigated the question as to whether the visibility of the roof of the CM is different in edentulous than in dentulous jaws. In two new studies, Iwanaga et al. examined this aspect anatomically and using CBCT. There were no differences in visibility of the CM roof between edentulous and dentulous sections [20, 21]. Oliveira-Santos et al. [12] presented similar results in a previous study.

One especially controversial approach to the visibility of the canal roof relates to the question as to whether gender and age are influential factors. Whilst Kubiliuset al [19]. and Oliveira-Santos et al. [12] could not determine any influence of gender and age on visibility, Iwanaga and Katafuchi et al. [20], Iwanaga and Shiromoto et al. [21] and Miles et al. [22] reported the contrary, albeit without having elucidated the cause. Kamrun et al. [23] noted that the reason could be because as age advances visibility decreases due to osteoporotic changes in the alveolar bone which reduces the visibility of mandibular canal. This thesis is further supported by the results of Iwanaga and Shiromoto et al. [21]. Their study shows that more females than males had osteoporotic mandibles; thus, when the canal roof cannot be seen on CBCT it is more likely to belong to osteoporotic than other mandibles groups.

By using both panoramic radiography and CBCT, Jung et al. [6] investigate whether the course of the mandibular canal impacts the visibility of the canal .The course of the mandibular canal was classified into four types: linear, elliptical, spoon-shaped, and turning curves. It was found that the visibility of the mandibular canal differed according to its course. The percentage of clearly visible mandibular canals was highest in spoon-shaped curves and lowest in linear curves.

In the present study, the two imaging methods were compared with regard to their diagnostic value for the detection of the roof of the canalis mandibulae. Statistically, a significant distinction was detected between the CBCT and the panoramic images in favour of the CBCT images (*p* < 0.05).

Furthermore, the thicknesses of the cortical bone of the respective regions P2-M3 were measured in the coronal CBCT sections buccally and lingually at the level of the canalis mandibulae. Here, the objective was to analyse the influence of the thickness of the cortical bone on the visibility of the canalis mandibulae in the PR images. No significant correlation could be ascertained between the visibility of the mandibular canal and the thickness of the cortical bone (*U* test, *p*≥0.05).

It may thus be concluded that the thickness of the cortical bone of the mandible exerts no influence on the visibility of the roof of canalis mandibulae in PR and CBCT images.

It is important to note that we were not able to find any analogous studies examining the visibility of the roof of the canalis mandibulae as a function of the thickness of the compacta.

## Conclusion

The present study concludes that, in contrast to PR images, the visibility of the roof of the mandibular canal is significantly higher in CBCT images. For a more advanced diagnosis of the canalis mandibulae roof in everyday practice, it would thus appear expedient, at least initially, to prioritise the CBCT. Ethical and radiobiological aspects must nonetheless be accounted for. Protection of the patient in accordance with the ALADA principle (as low as diagnostically acceptable) is imperative [24]. Furthermore, pursuant to § 8 of the Radiation Protection Act, the benefits to health must invariably prevail over the risks of radiation [25]. Here, CBCT technology remains ancillary to conventional X-ray technology.

Furthermore, the thickness of the cortical bone of the mandible was found to exert no impact on the visibility of the roof of the canalis mandibulae.

Additional studies are required to investigate potential influencing factors on the visibility of the canal roof. To extend scientific knowledge acquired thus far, subsequent research should also further investigate the influence of jaw width on the visibility of apical whitening.

## Data Availability

The original datasets analysed in the current study are available from Dr. Ali Reza Ketabi on reasonable request.

## Abbreviations

CBCT: Cone-beam computed tomography
CT: Computertomographie
PR: Panoramic radiograph
CM: Canalis mandibulae
N: Nervus
FOV: Field of view
P2: Second premolar
M1: First molar
M2: Second molar
M3: Third molar
